# Hémoglobinurie chez l’enfant à Ouagadougou: prise en charge hospitalière et pronostic à court terme

**DOI:** 10.11604/pamj.2019.34.165.14729

**Published:** 2019-11-26

**Authors:** Hamidou Savadogo, Gérard Coulibaly, Viviane Bandaogo, Aïssata Kaboré, Lassina Dao, Sonia Kaboret, Solange Odile Ouédraogo-Yugbaré, Fla Kouéta, Diarra Yé

**Affiliations:** 1 Service de Pédiatrie Médicale, Centre Hospitalier Universitaire Pédiatrique Charles-de-Gaulle, Ouagadougou, Burkina Faso; 2 Service de Néphrologie et Hémodialyse, Centre Hospitalier Universitaire Yalgado Ouédraogo, Ouagadougou, Burkina Faso

**Keywords:** Hémoglobinurie, insuffisance rénale, anémie, paludisme, déficit en G6PD, Hemoglobinuria, renal failure, anemia, malaria, G6PD deficiency

## Abstract

**Introduction:**

L’objectif de ce travail était d’analyser les aspects épidémiologiques, diagnostiques, thérapeutiques et évolutifs de l’hémoglobinurie chez l’enfant au CHU Pédiatrique Charles-De-Gaulle de Ouagadougou.

**Méthodes:**

Il s’est agi d’une étude transversale à visée descriptive sur la période allant du 01 juillet au 31 décembre 2014. Ont été inclus dans l’étude, tous les enfants âgés de zéro à 15 ans hospitalisés dans le Service de Pédiatrie Médicale du CHU Pédiatrique Charles-de-Gaulle, chez qui une hémoglobinurie macroscopique a été diagnostiquée pendant la période de l’étude.

**Résultats:**

Trente-huit patients ont été inclus dans l’étude. La fréquence hospitalière de l’hémoglobinurie était de 1,9%. L’âge moyen des patients était de 80,8 ± 44,1 mois (extrêmes = 21 et 168). Il s’agissait de 23 garçons (60,5%) et 15 filles (39,5%). Les principaux signes cliniques étaient: la fièvre (86,8%), les urines foncées « coca cola » (86,8%), la pâleur (63,2%), l’hépatomégalie (50%). Le débit de filtration glomérulaire DFG était inférieur à 80 mL/min/1,73m^2^ chez 23 patients (69,7%); 21 patients avaient un déficit en G6PD. Les principales étiologies présumées de l’hémoglobinurie étaient: le paludisme grave, les infections bactériennes et virales, le déficit en G6PD, la fièvre bilieuse hémoglobinurique. Les traitements administrés étaient: les dérivées de l’artémisinine, les antibiotiques et les antipyrétiques. Le recours à la dialyse a été nécessaire chez un patient.

**Conclusion:**

L’hémoglobinurie est un symptôme qui pose surtout un problème de diagnostic étiologique dans notre contexte. Sa gravité réside dans le fait qu’elle peut provoquer une IRA sévère.

## Abstract (en)

**Introduction:**

The purpose of this study was to analyze the epidemiological, diagnostic, therapeutic and evolutionary features of hemoglobinuria in children hospitalized in the Pediatric University Hospital Charles de Gaulle, Ouagadougou.

**Methods:**

We conducted a cross-sectional descriptive study over the period 01^st^ July-31^st^ December 2014. All children aged 0-15 years hospitalized in the Department of Medical Pediatrics of the Pediatric University Hospital Charles de Gaulle and diagnosed with macroscopic hemoglobinuria during the study period were enrolled.

**Results:**

Thirty-eight patients were included in the study. Hospitalization rate for hemoglobinuria was 1.9%. The average age of patients was 80.8 ± 44.1 months (ranging from 21 to 168). The study involved 23 boys (60.5%) and 15 girls (39.5%). The major clinical signs were: fever (86.8%), dark urines like « coca cola » (86.8%), pallor (63.2%), hepatomegaly (50%). Glomerular filtration flow was less than 80 mL/min/1.73m^2^ in 23 patients (69.7%); 21 patients had Glucose-6-phosphate dehydrogenase (G6PD) deficiency. The main suspected causes of hemoglobinuria were: severe malaria, bacterial and viral infections, G6PD deficiency, biliary haemoglobinuric fever. Treatments included: artemisinin derivatives, antibiotics and antipyretics. One patient underwent dialysis.

**Conclusion:**

Hemoglobinuria is a symptom mainly causing diagnostic problems in our context. It is a severe disorder which can result in acute renal failure (ARF).

## Introduction

L’hémoglobinurie s’accompagne d’une coloration foncée des urines d’aspect «coca-cola». Elle est observée surtout au cours des pathologies hémolysantes (infectieuse, immunologique, congénitale, allergique, traumatique, intoxication) [1]. Les cas d’hémoglobinurie décrits dans la littérature sont surtout secondaires à la fièvre bilieuse hémoglobinurique, au déficit en hémoglobinurie paroxystique nocturne (HPN), aux origines toxiques et médicamenteuses. Dans nos contrées, l’hémoglobinurie est de plus en plus rencontrée en pratique médicale surtout pendant la période de l’hivernage où sévit le paludisme. Aussi, avec l’avènement de la chloroquino-résistance et l’utilisation de la quinine comme médicament de choix dans le traitement du paludisme grave, des cas d’hémoglobinurie sont de plus en plus décrits en milieu pédiatrique [2]. Son incidence hospitalière est comprise entre 17,2 et 19,1% en milieu pédiatrique en Afrique de l’Ouest [3, 4]. C’est un symptôme potentiellement grave à cause de son évolution fréquente vers l’insuffisance rénale d’où la nécessité d’un diagnostic précoce pour une prise en charge adéquate des patients. C’est pourquoi nous nous sommes proposé à travers cette étude, de faire le point sur les hémoglobinuries chez les enfants de zéro à 15 ans, hospitalisés dans le service de pédiatrie médicale du Centre Hospitalier Universitaire Pédiatrique Charles-De-Gaulle (CHUP-CDG). Les résultats fournis par l’étude pourraient contribuer à l’amélioration de la prise en charge des enfants présentant une hémoglobinurie dans notre contexte de travail.

## Méthodes

Il s’est agi d’une étude transversale à visée descriptive. Elle a concerné la période allant du 1er juillet au 31 décembre 2014 soit six mois. L’étude a eu pour cadre le service de pédiatrie médicale du CHUP-CDG de Ouagadougou. Ont été inclus dans l’étude, tous les enfants âgés de 0 à 15 ans hospitalisés dans le service pendant la période de l’étude, chez qui une hémoglobinurie a été diagnostiquée. Il s’est agi d’un échantillonnage de convenance. Nous avons recueilli à l’aide d’une fiche de collecte individuelle les données sociodémographiques concernant les patients, les antécédents pathologiques, les signes cliniques et paracliniques, les modalités thérapeutiques et évolutives. En l’absence d’un comité d’éthique au sein du CHUP-CDG, l’autorisation de la direction générale du CHU a été obtenue avant le début de la collecte. Le consentement éclairé des parents et l’assentiment de l’enfant (lorsque celui-ci était en mesure de le faire) ont été recueillis avec respect strict du secret médical.

Les données ont été saisies et traitées sur micro-ordinateur à l’aide des logiciels SPSS 20 et CSPro 6.0. Les variables analysées étaient les données sociodémographiques (âge, sexe, scolarité, lieu de résidence, profession des parents), les signes cliniques et paracliniques, les modalités thérapeutiques et évolutives. Les variables continues ont été exprimées par leur moyenne ± déviation standard et les variables discontinues par les fréquences absolue et relative. Les patients ont été suivis pendant trois mois afin d’apprécier l’évolution de de la fonction rénale. Nous avons défini de façon opérationnelle

***L’hémoglobinurie:*** dans notre étude nous avons retenu l’hémoglobinurie macroscopique, sur la base de la coloration foncée couleur «coca cola» des urines en dehors de toute cause suggérant une myoglobinurie et d’hématurie.

***Le déficit d’activité de la Glucose-6-Phosphate Déshydrogénase (G6PD):*** il a été diagnostiqué par le dosage de l’activité en G6PD de l’hémoglobine. Une baisse de l’activité de la G6PD par rapport à la normale (normes du laboratoire) en dehors de tout épisode aigu d’hémolyse était considérée comme un déficit.

***Le paludisme grave:*** il a été défini par la présence de trophozoïtes de *Plasmodium falciparum* à l’examen microscopique du sang d’un patient, associée à au moins un des critères de gravité définis par l’Organisation Mondiale de la Santé (OMS) [2, 5].

***La fièvre bilieuse hémoglobinurique:*** elle a été définie par l’association d’une hémoglobinurie macroscopique fébrile avec une goutte épaisse négative ou positive et une notion de traitement antérieur par la quinine, la méfloquine ou l’halofantrine [2]. Nous avons en outre évoqué la fièvre bilieuse hémoglobinurique devant une notion de traitement fréquent et récent à la quinine.

### Anémie

Pour notre étude nous avons considéré un taux d’hémoglobine <11g/dL. En fonction du taux d’hémoglobine, l’anémie a été considérée [6]: 1) légère lorsque le taux d’hémoglobine est compris entre 10 et 11g/dL; 2) modérée lorsque le taux d’hémoglobine est compris entre 7 et 9g/dL; 3) sévère lorsque le taux d’hémoglobine est strictement inférieur à 7g/dL.

### Insuffisance rénale

Nous l’avons définie par un débit de filtration glomérulaire (DFG) inférieur à 80 mL/min/1,73 m^2^ de surface corporelle. Le DFG a été estimé par la clairance de la créatinine calculée grâce à la formule de Pottel [7]:

DFG=107,3/[Créatininémie(mg/L)/Q] avec Q=0,027*âge+0,2329. L’insuffisance rénale a été considérée comme aiguë en cas de normalisation secondaire de la fonction rénale en moins de trois mois. Elle était chronique si la fonction rénale restait altérée au-delà de trois mois d’évolution.

## Résultats

### Fréquence de l’hémoglobinurie

**Figure 1:**
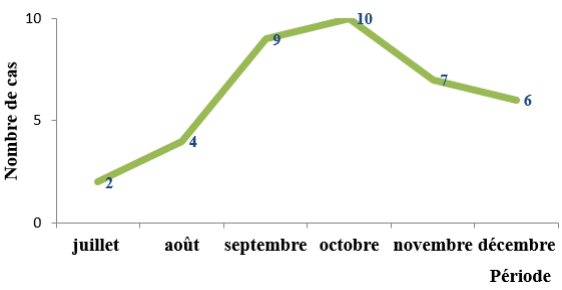
Fréquence mensuelle de l´hémoglobinurie

Trente-huit patients ont été inclus dans l’étude durant la période de six mois soit une moyenne de 6,3 cas par mois. Ces patients représentaient 1,9% de l’ensemble des patients (n = 1993) hospitalisés dans le service de pédiatrie médicale du CHUP-CDG durant la période d’étude. La Figure 1 montre la variation du nombre de cas d’hémoglobinurie observés en fonction du mois.

### Caractéristiques sociodémographiques

La moyenne d’âge de nos patients était de 80,8 ± 44,1 mois. Le sex ratio était de 1,5. Les enfants d’âge compris entre 60 et 120 mois représentaient 42,1% de l’effectif global (Tableau 1). Concernant la profession des parents, les pères étaient cultivateurs (agriculteurs) dans 50% des cas et les mères ménagères dans 81,6% des cas. Les autres parents étaient essentiellement des fonctionnaires et des salariés du privé. Le lieu de résidence était urbain dans 57,9% des cas, semi-urbain dans 26,3% et rural dans 15,8% des cas.

**Tableau 1:** Répartition des patients en fonction de la classe d’âge et le sexe (n = 38)

Classes d'âge (mois)	Sexe		Total n (%)
	Filles	Garçons	
0 – 59	7 (18,4)	6 (15,8)	13 (34,2)
60 – 119	5 (13,2)	11 (28,9)	16 (42,1)
120 -180	3 (7,9)	6 (15,8)	9 (23,7)
Total	15 (39,5)	23 (60,5)	38 (100)

### Antécédents des patients

L’interrogatoire a permis de noter: 1) un antécédent de prise de produits traditionnels dans 5,3% des cas; 2) un antécédent d’émission d’urines d’aspect « coca-cola » dans 7,9% des cas; 3) un syndrome drépanocytaire majeur SS; 4) vingt-et-neuf patients soit 76,3% des cas étaient à jour de leurs vaccinations selon le programme élargi de vaccination; 5) aucun patient n’a rapporté un antécédent familial d’émission d’urine d’aspect « coca-cola».

### Motifs d’admission

Les principaux motifs de consultation ou de référence étaient: la fièvre (29 cas soit 76,3%), l’hémoglobinurie (23 cas soit 60,5%), la pâleur (15 cas soit 39,5%), les vomissements (14 cas soit 36,8%) et les douleurs abdominales (10 cas 26,3%).

### Signes cliniques et paracliniques

Les principaux signes cliniques étaient: la pâleur cutanéomuqueuse (24 cas soit 63,1%), l’ictère (17 cas soit 44,7%), l’hépatomégalie (19 cas soit 50%), la splénomégalie (16 cas soit 42,1%) et l’altération de l’état général (15 cas soit 39,5%). Sur le plan paraclinique, des examens biochimiques (Tableau 2) ainsi qu’hématologiques, immunologiques, parasitologiques et bactériologiques ont été effectués. La créatininémie était élevée dans 42,4% des cas. L’azotémie était disponible chez 29 patients (76% des patients). Elle était élevée chez 11 patients soit 37,9% des cas.

**Tableau 2:** Résultats d’analyses biochimiques sanguines réalisées chez les patients

	n (%)	Moyenne ± DS	Extrêmes
Urée (mmol/L)	29 (76)	11,1 ± 11,14	1,6 - 51,6
Créatinine (µmol/L)	33 (86,8)	146,9 ± 217,6	4,3 – 962
Sodium (mEq/L)	21 (55,3)	135,7 ± 4,8	127 – 146
Potassium (mEq/L)	21 (55,3)	4,1 ± 0,5	3,30 - 5,1
Calcium (mEq/L)	25 (65,8%)	2,3 ± 1,1	1,64 - 7,7
Bicarbonate (mmol/L)	5 (13,6%)	17,6 ± 5,4	9,00 – 23
Magnésium (mEq/L)	21 (55,3%)	0,7 ± 0,2	0,27 - 1,4
Protides totaux (g/L)	22 (57,9%)	63,5 ± 12,9	40- 90
Glucose (mmol/L)	27 (71,1%)	4,7 ± 1,3	2,10 - 7,2
ASAT (UI/L)	9 (23,7)	151,2 ± 145,5	29 – 445
ALAT (UI/L)	9 (23,7)	164,5 ± 194,5	18 – 653
Bilirubine totale (µmol/L)	23 (60,5)	70,3 ± 185,3	7,3 - 903,5
Bilirubine conjuguée (µmol/L)	23 (60,5)	47,3 ± 135,7	3,3 - 653,8

N : nombre de patients chez qui l’analyse biochimique a été réalisée

La goutte épaisse était positive à *Plasmodium falciparum* chez 60,5% des patients. Le dosage de l’activité de la G6PD a été possible chez 25 patients soit 65,8% des cas. Vingt-un patients soit 84% des patients ayant réalisé un dosage de l’activité de la G6PD étaient déficitaires en G6PD. La sérologie du virus de l’immunodéficience humaine (VIH) a été effectuée chez six patients et était négative dans tous les cas. La sérologie de l’hépatite était positive chez deux patients. Un patient était atteint de la dengue. À l’hémogramme, une hyperleucocytose dans 24 cas soit 63,1%. L’échographie abdominale a été pratiquée chez 13 patients. La taille moyenne du rein gauche des 13 patients était de 100,2 ± 4,8 mm (extrêmes = 68 et 133), celle du rein droit de 87,8 ± 6,2 mm (extrêmes = 54 et 127).

### Étiologies

Les principales pathologies présumées associées à l’hémoglobinurie dans notre étude étaient:

Le paludisme grave: il a été évoqué chez 35 patients soit 92,1% des cas. La goutte épaisse était positive chez 23 patients soit 60,5% des cas. Parmi les cas de paludisme confirmés, 14 soit 36,8% de tous les patients étaient de sexe masculin et neuf soit 23,7% étaient de sexe féminin. L’âge moyen de ces patients était de 67,4±41,7 mois (extrêmes = 21 et 168).

Le déficit d’activité de la G6PD: il a été confirmé chez 21 patients soit 55,3% des cas. Parmi ces patients 18 soit 47,3% de l’ensemble des patients, avaient une infection bactérienne probable et un paludisme grave.

La fièvre bilieuse hémoglobinurique: elle a été évoquée chez 15 patients soit 39,5% des cas. Une notion de de traitement fréquent et récent à base de quinine avait été rapportée chez ces patients. Ils étaient majoritairement de sexe masculin (26,3%). Leur âge moyen était de 78,6 ± 49,4 mois (extrêmes = 24 et 168). La goutte épaisse était positive chez six de ces patients soit 15,8% des cas.

Les infections bactériennes: elles avaient été évoquées chez 19 patients soit 50% des cas, sur la base d’une hyperleucocytose. Les sites des infections bactériennes étaient présumés urinaire dans 15 cas (39,5%), broncho-pulmonaire dans trois cas (7,9%) et méningé dans un cas (2,6%).

### Complications

Les complications de l’hémoglobinurie étaient essentiellement l’anémie sévère et l’insuffisance rénale aiguë. Une anémie sévère a été diagnostiquée chez 17 patients soit 44,7% des cas, dont neuf garçons (23,7%) huit filles (21%). L’âge moyen de ces patients était de 68,5 ± 43,7 mois (extrêmes = 24 et 168). Leur taux d’hémoglobine moyen était de 4,0 ± 1,1g/dL (extrêmes = 2,3 et 5,7). L’estimation du DFG par la formule de Pottel a révélé que 23 patients (60,5%) dont 15 garçons (39,5%) avaient une insuffisance rénale aiguë. L’âge moyen de ces patients était de 84,1 ± 51,2 mois (extrêmes = 21 et 168). La créatininémie moyenne de ces patients était de 197,1 ± 245,1 µmol/L (extrême = 38 et 962).

### Modalités thérapeutiques

Quinze patients soit 39,5% des cas avaient reçu de la quinine avant l’admission au service tandis que 10 autres (26,3%) avaient reçu de la ceftriaxone et six (15,8%) de la sulfadoxine-pyriméthamine. En cours d’hospitalisation des antipaludiques ont été utilisés chez 36 patients soit 94,7% des cas. Il s’est agi de: 1) l’artésunate injectable à la dose de 2,4 mg/kg a été utilisée dans 22 cas soit 61,1%; 2) l’artéméther injectable a été administré à 14 patients soit 38,9% des cas.

Une antibiothérapie a été administrée à 32 patients soit 84,2% des cas. Les antibiotiques les plus utilisés étaient la ceftriaxone (32 cas soit 84,2%), la gentamicine (14 cas; 36,8%), l’association amoxicilline-acide clavulanique (10 cas; 26,3%). Le traitement anti-anémique a consisté en une transfusion de concentrés de globules rouges (CGR) isogroupe isorhésus Elle a été effectuée chez 27 patients (71% des cas) au cours de leur hospitalisation. Le taux d’hémoglobine était inférieur à 6 g/dL dans tous ces cas. Le recours à l’hémodialyse a été fait chez un garçon âgé de 12 ans qui avait une insuffisance rénale sévère anurique (créatininémie à 1593µmol/L au quatrième jour d’hospitalisation). Trois séances d‘hémodialyse ont été nécessaires.

### Évolution

La durée moyenne d’hospitalisation était de 9,8 ± 6,5 jours (extrêmes = 2 et 25). Trois modes de sortie après l’hospitalisation étaient notés : l’évasion (deux cas soit 5,3%), le décès (un cas par défaillance multiviscérale soit 2,6%), la rémission de l’hémoglobinurie (35 cas soit 92,1%). Parmi les 35 cas de rémission, 30 patients soit 78,9% des cas ont été suivis et cinq étaient perdus de vue soit 13,2% des cas. Tous les patients en rémission avaient un taux d’hémoglobine minimum à huit à leur sortie. Les créatininémies de contrôle à la sortie, à un mois et à trois mois étaient normales chez les patients qui ont été suivis (Tableau 3). Une notion de récidive de l’hémoglobinurie a été rapportée chez sept patients soit 18,4% des cas.

**Tableau 3:** Répartition des patients en fonction de la créatininémie de contrôle réalisée à la sortie, à un mois et à trois mois

Créatininémie de contrôle	Normale	Élevée
A la sortie	35	0
A 1 mois	30	0
A 3 mois	24	0

## Discussion

L’absence de financement a constitué une difficulté à la réalisation de certains examens paracliniques du fait de leur coût relativement élevé pour les parents. De même certains examens paracliniques n’étaient pas disponibles dans notre contexte (test de Coombs, dosage du pyruvate kinase). De plus, l’étude étant hospitalière, nos résultats ne sauraient être extrapolés à la population générale. En dépit de ces limites cette étude nous a permis d’obtenir des résultats que nous avons comparés avec les résultats d’autres auteurs.

La fréquence hospitalière de l’hémoglobinurie était de 38 cas en six mois dans notre étude avec une incidence de 1,9%. Une incidence de 3,3% a été rapportée dans une étude de l’OMS, réalisée en milieu pédiatrique, dans dix pays d’Afrique dont le Nigéria sur le paludisme grave [8]. D’autres auteurs ont trouvé par contre des incidences plus élevées. C’est le cas de Verma et coll en Inde (19,6%), Ajetunmobi *et al.* à Ibadan au Nigéria (19,1%), de Gbadoé au Togo (17,2%) [3, 4, 9]. Gobbi au Burundi notait quant à lui une incidence hospitalière inférieure à la nôtre (0,9%) [10]. De telles différences pourraient être liées à deux faits: la petite taille de notre population d’étude rapportée à l’ensemble des hospitalisés durant la période de l’étude (n = 1993); et le fait que les patients inclus dans les autres études étaient uniquement des cas de paludisme grave ou de fièvre bilieuse hémoglobinurique.

La majorité des cas d’hémoglobinurie a été enregistrée durant les mois de septembre, octobre et novembre. Verma *et al.* en Inde faisaient le même constat [9]. Cette période, qui correspond à la fin de l’hivernage au Burkina Faso tout comme en Inde, est une période de forte transmission du paludisme grave qui était présumé être la principale cause d’hémoglobinurie dans notre série. La chimioprévention du paludisme saisonnier, préconisée par l’OMS, ne s’avèrerait pas seulement nécessaire pour les moins de cinq ans mais aussi pour tous les enfants en zone d’endémie palustre.

L’âge moyen des patients dans notre série était de 80,8 ± 44,1 mois (6,7 ± 3,7 ans) avec une prédominance des enfants de 60 à 119 mois (42,1%). Ce résultat corrobore ceux de Lalya et coll à Cotonou, de Mbanya *et al.* et de Verma *et al.* en Inde qui avaient observé des âges moyens respectifs de 7 ans 1 mois, 86,7 ± 22,4 mois et 6 ans avec une prédominance des enfants de 60 à 120 mois [9, 11, 12]. Il était différent de ceux de Ajetunmobi et coll à Ibadan au Nigéria ainsi que de Kunuanunua *et al.* qui notaient dans leur série une prédominance des enfants de moins de cinq ans [3, 13]. Les patients de sexe masculin étaient les plus représentés dans notre étude avec 60,5% des cas. Nos résultats sont comparables à ceux de Nacoulma *et al.* au Burkina Faso, Ajetunmobi *et al.* à Ibadan au Nigéria qui notaient une prédominance masculine dans respectivement 69% et 62,7% des cas [3, 14]. La fréquence élevée du déficit d’activité de la G6PD pourrait expliquer la prédominance masculine dans notre étude. Le déficit en G6PD est une maladie à transmission génétique récessive liée au chromosome X qui touche majoritairement les hommes dits homozygotes [15-17].

Les urines foncées d’aspect «coca-cola» étaient le motif de référence le plus fréquent dans notre série avec 60,5% des cas. Il s’agit d’un signe de gravité des pathologies hémolytiques. Elle constitue aussi un signe de gravité du paludisme [10, 18]. En outre, l’apparition d’urines foncées d’aspect «coca-cola» fréquemment associée à une anurie ou à une oligurie pourrait être à l’origine d’une angoisse chez le patient et/ou chez ses parents qui consulteraient le plus rapidement possible dans un centre de santé. Néanmoins, les cas «mineurs» avec des urines peu colorées pour lesquels il n’y aura pas de consultation contribuent à une sous-estimation de la fréquence de l’hémoglobinurie. La pâleur conjonctivale a été observée chez 24 patients soit 63,1% des cas. Des études effectuées sur la fièvre bilieuse hémoglobinurique au Burkina Faso, en 2009 par Nacoulma *et al.* en 2014 par Savadogo notaient aussi une fréquence élevée de la pâleur conjonctivale [14, 19]. La fréquence élevée de la pâleur s’explique par l’installation d’une anémie consécutive à l’hémolyse.

L’hépatomégalie était le signe physique le plus observé avec 19 cas (50%), suivi de la splénomégalie observée dans 17 cas (42,1%). Tran et cool dans leur étude notaient aussi l’hépatomégalie dans 25 cas (50%), la splénomégalie dans 15 cas (34%) et une hépatosplénomégalie dans 9 cas (18%) [20]. La séquestration splénique d’hématies pourrait expliquer la splénomégalie dans notre série tout comme dans celle de Tran. L’hépatomégalie quant à elle, pourrait être secondaire soit à un hyperfonctionnement des hépatocytes liée leur diminution suite à une lyse importante ou à une infection des hépatocytes par des micro-organismes (bactériens, viraux, parasitaires). Faute de brassards adaptés, la tension artérielle n’a été mesurée que chez 22 patients. Un seul cas d’hypertension artérielle systolique transitoire (4,5%) a été observé. Ajetunmobi *et al.* observaient une élévation transitoire de la pression artérielle systolique chez 2,3% de leurs patients [3].

La créatininémie était élevée dans 42,4% des cas. La formule de Pottel nous a permis de calculer le débit de filtration glomérulaire chez tous les patients chez qui la créatininémie avait pu être mesurée. Cette formule, bien que ne prenant pas en compte la taille comme dans la formule de Schwartz, est fiable pour l’estimation du DFG chez l’enfant [21]. Néanmoins il ne faudrait pas perdre de vue que ces deux formules sont a priori utilisées pour les IRC [7].

Dans notre série 60,5% des patients ont présenté une insuffisance rénale aiguë. Daubrey et coll en Côte d’Ivoire et Kunuanunua *et al.* à Kinshasa avaient trouvé des fréquences plus élevées de 90% et 90,1% dans leurs études [13, 18]. En revanche, la fréquence de l’insuffisance rénale notée dans notre étude, est supérieure à celle de Balaka *et al.* au Togo qui avaient observé une fréquence d’IRA post-hémolytique de 35,1% chez des patients déficients en G6PD [22]. Le fait d’avoir défini l’insuffisance rénale par une simple élévation de la créatininémie au-dessus de la normale, peut surestimer (repas hyperprotéique, race noire, l’obésité) ou sous-estimer (malnutrition) le nombre de cas d’insuffisants rénaux dans les études de Daubrey, Kunuanunua et Balaka. Cela pourrait expliquer la différence des résultats avec les nôtres. La toxicité de l’hémoglobine pour le tube rénal est connue. Le mécanisme de l’IRA bien que mal élucidé serait essentiellement une nécrose tubulaire (toxicité directe, micro-obstruction tubulaire). L’atteinte glomérulaire directe secondaire à une l’anémie hémolytique et/ou à une chute du débit sanguin rénal serait un autre mécanisme [13].

Le dosage de l’activité de la G6PD chez 25 patients avait retrouvé 21 déficitaires (84%). Tran *et al.* au Vietnam, Balaka *et al.* au Togo ainsi que Youl au Burkina Faso ont rapporté respectivement 54%, 32,1% et 11,69% de patients présentant un déficit en G6PD dans leurs études [20, 22, 23]. Le déficit en G6PD est une cause fréquente d’hémolyse intravasculaire massive. C’est une maladie génétique à transmission gonosomique liée au chromosome X [16, 24]. Sa répartition géographique est grossièrement superposable à celle du paludisme et de la drépanocytose augmentant ainsi le risque d’épisodes hémolytiques dans ces régions [17, 24]. Cependant, malgré ces conséquences cliniques variables et potentiellement sévères, cette pathologie est très peu connue de nos populations. Son dépistage néonatal, bien qu’essentiel dans notre contexte n’est pas encore réalisé. La mise en place d’un programme national de dépistage du déficit d’activité de la G6PD serait d’un grand apport pour le clinicien. En effet le dépistage néonatal du déficit en G6PD a un double bénéfice. Il permettra non seulement d’instaurer précocement une prophylaxie anti-infectieuse et anti-oxydante, mais aussi d’éduquer les parents.

La majorité des patients dans notre étude avaient reçu un traitement antipaludique à base d’artésunate injectable ou d’artéméther injectable. Cela s’explique par le fait que selon les nouvelles directives de prise en charge du paludisme grave, l’artésunate injectable est la molécule de choix suivi de l’artéméther injectable et enfin de la quinine [3, 5, 25]. Ces molécules sont incriminées dans la genèse de la fièvre bilieuse hémoglobinurique [18]. Les antipaludiques administrés avant l’hospitalisation (quinine, sulfadoxine-pyriméthamine) ainsi que le déficit d’activité de la G6PD chez certains de nos patients étaient sans doute des facteurs favorisants dans la survenue de l’hémoglobinurie. La durée moyenne de séjour hospitalier relativement courte dans notre série (9,8 ± 6,5 jours). Cela pourrait s’expliquer par l’amélioration rapide des complications (anémie, insuffisance rénale aiguë) sous traitement médical. L’épuration extra-rénale était nécessaire chez un seul patient.

L’évolution était favorable chez la majorité des patients (94,7%) et fatale chez deux patients (5,3%). Balaka au Togo a noté une évolution favorable dans 80,7% des cas et fatale dans 19,2% des cas [22]. Le bon pronostic pourrait s’expliquer par une prise en charge rapide et adéquate des patients. Des cas de récidive avaient été rapportés chez sept patients soit 14,2% des cas. Parmi ces patients, cinq étaient déficitaires en G6PD. La haute susceptibilité aux infections des patients atteints d’une enzymopathie telle le déficit en G6PD et/ou d’une hémoglobinopathie [17, 26, 27] ainsi que le caractère endémique du paludisme dans notre contexte pourraient expliquer les cas de récidive dans notre série.

## Conclusion

Cette étude nous a révélé que l’hémoglobinurie est un symptôme relativement fréquent chez l’enfant dans notre contexte. L’infection à *Plasmodium falciparum* était la première cause d’hémoglobinurie et était généralement associée à d’autres facteurs telles les infections bactériennes et virales, le déficit en G6PD. Elle nous a montré que le déficit en G6PD est également une cause fréquente d’hémoglobinurie. Il serait souhaitable que des études multicentriques soient menées afin de déterminer la prévalence du déficit en G6PD à l’échelle nationale ainsi que son association à d’autres maladies. En outre, l’instauration d’un dépistage néonatal de cette enzymopathie et des hémoglobinopathies s’avère nécessaire dans notre pays, car cela permettra au clinicien de disposer d’un diagnostic étiologique en cas d’hémolyse, mais aussi et surtout d’avoir une action préventive en informant les parents du nouveau-né sur les précautions à prendre afin de prévenir les crises hémolytiques.

### Etat des connaissances actuelles sur le sujet

- Le déficit en G6PD est une cause fréquente d’hémoglobinurie;
- L’hémoglobinurie peut se compliquer d’insuffisance rénale aiguë.

### Contribution de notre étude à la connaissance

- Le paludisme peut être responsable d’hémoglobinurie dans notre contexte;
- L’évolution de l’insuffisance rénale aiguë causée par l’hémoglobinurie est souvent favorable.

## Conflits d’intérêts

Les auteurs ne déclarent aucun conflit d’intérêts.
